# TAp63 determines the fate of oocytes against DNA damage

**DOI:** 10.1126/sciadv.ade1846

**Published:** 2022-12-21

**Authors:** Yi Luan, Seok-Yeong Yu, Amirhossein Abazarikia, Rosemary Dong, So-Youn Kim

**Affiliations:** ^1^ Olson Center for Women’s Health, Department of Obstetrics and Gynecology, College of Medicine, University of Nebraska Medical Center, Omaha, NE, USA.; ^2^ Fred and Pamela Buffett Cancer Center, University of Nebraska Medical Center, Omaha, NE, USA.

## Abstract

Cyclophosphamide and doxorubicin lead to premature ovarian insufficiency as an off-target effect. However, their oocyte death pathway has been debated. Here, we clarified the precise mechanism of ovarian depletion induced by cyclophosphamide and doxorubicin. Dormant oocytes instead of activated oocytes with high PI3K activity were more sensitive to cyclophosphamide. Checkpoint kinase 2 (CHK2) inhibitor rather than GNF2 protected oocytes from cyclophosphamide and doxorubicin, as cyclophosphamide up-regulated p-CHK2 and depleted primordial follicles in *Abl1* knockout mice. Contrary to previous reports, TAp63 is pivotal in cyclophosphamide and doxorubicin-induced oocyte death. Oocyte-specific *Trp63* knockout mice prevented primordial follicle loss and maintained reproductive function from cyclophosphamide and doxorubicin, indicated by undetectable levels of BAX and cPARP. Here, we demonstrated that TAp63 is fundamental in determining the signaling of oocyte death against DNA damage. This study establishes the role of TAp63 as a target molecule of adjuvant therapies to protect the ovarian reserve from different classes of chemotherapy.

## Teaser

Understanding the specific oocyte death pathway by each chemotherapeutic agent will help develop fertoprotectants against it.

## INTRODUCTION

Cancer survivors look forward to long-term survival and high quality of life after treatment. Although advanced cancer therapies have remarkably improved the life expectancy of cancer survivors, the treatment itself increases the risk of reproductive insufficiency. Because women are born with a finite number of oocytes that cannot be regenerated during a woman’s reproductive life, an exhausting follicular pool accelerates the onset of menopause irrespective of age, resulting in endocrine dysfunction and infertility. This becomes a critical status in prepubertal girls and premenopausal women (*1*, *2*). Therefore, it is necessary to elucidate a precise mechanism of ovarian follicle death in the ovary following the administration of chemotherapy.

Cyclophosphamide (CPA) is an alkylating agent for cancer patients with leukemia, lymphomas, breast cancer, and bone and soft tissue sarcomas (*3*). CPA induces apoptosis in proliferating tumor cells by alkylating DNA at the N-7 position of guanine, causing DNA damage in the form of double-strand breaks, interstrand and intrastrand DNA cross-links, and DNA-protein cross-links (*4*). Exposure to CPA was identified as a significant risk factor for acute ovarian failure in childhood cancer survivors through follicle depletion and ovarian hormone deficiency (*5*, *6*). It is known that patients with cancer treated with CPA present lower serum anti–Müllerian hormone (AMH) and fewer antral follicles, offering a higher risk of amenorrhea or oligomenorrhea (*7*, *8*). Although a burnout theory was proposed to explain the mechanism of CPA-induced primordial follicle loss (*9*), most of the studies demonstrated that CPA directly destroys oocytes of primordial follicles through the apoptotic pathway because the oocytes in primordial follicles have a high sensitivity to DNA damage (*10*–*12*). However, the precise mechanisms of oocyte death by DNA damage in primordial follicles require more understanding.

Previous studies proved that irradiation and cisplatin (CDDP) distinctly induce oocyte death of primordial follicles, implying that oocytes initiate different apoptotic pathways depending on the chemotherapeutic agents. Although the role of TAp63 as the main factor or as a cofactor appears unclear, the relevance of TAp63 in the oocytes of primordial follicles is prevalent regardless of the distinct upstream mechanisms (*11*). TAp63 is phosphorylated by kinases of Casein kinase 1 (CK1) and CHK2 at four consecutive sites (S585, S588, S591, and T594) upon DNA damage induced by irradiation or CDDP (*13*, *14*). Primarily, the phosphorylation of TAp63 at S591 determines tetramerization and induces transcription of *puma* and *noxa* to cause apoptosis of DNA-damaged primordial follicle oocytes (*15*, *16*). However, the precise downstream mechanisms of oocyte death by CPA remains to be further determined. A previous study proposed that inhibition of the ABL-TAp63 pathway using an ABL kinase inhibitor protects oocytes from CPA-induced death (*17*). In addition, another study suggested that oocyte death by CPA is not related to the presence of TAp63 (*18*). Thus, it was necessary to clarify whether TAp63 is a key molecule in determining the fate in oocytes of primordial follicles regardless of the class of chemotherapeutic agents.

We previously demonstrated that CPA directly destroys dormant oocytes of primordial follicles without increasing the number of growing follicles (*12*). Here, we investigated the mechanisms of ovarian reserve loss by CPA using oocyte-specific Pik3ca^*^, *Abl1*, and *Trp63* mouse lines. We also examined the gonadotoxicity of doxorubicin (DOXO), a topoisomerase inhibitor, using mouse lines to uncover the oocyte death pathway by a different class of chemotherapeutic agent. We found that ABL is dispensable for CPA-induced primordial follicle loss, while TAp63 is a critical molecule to determine the fate of oocytes in primordial follicles against CPA and DOXO. Thus, this study demonstrates the essential role of TAp63 as a target molecule of adjuvant therapies to protect the ovarian reserve from chemotherapy and radiation therapy.

## RESULTS

### CPA directly destroys oocytes of primordial follicles in dormant status

**Fig. 1.**
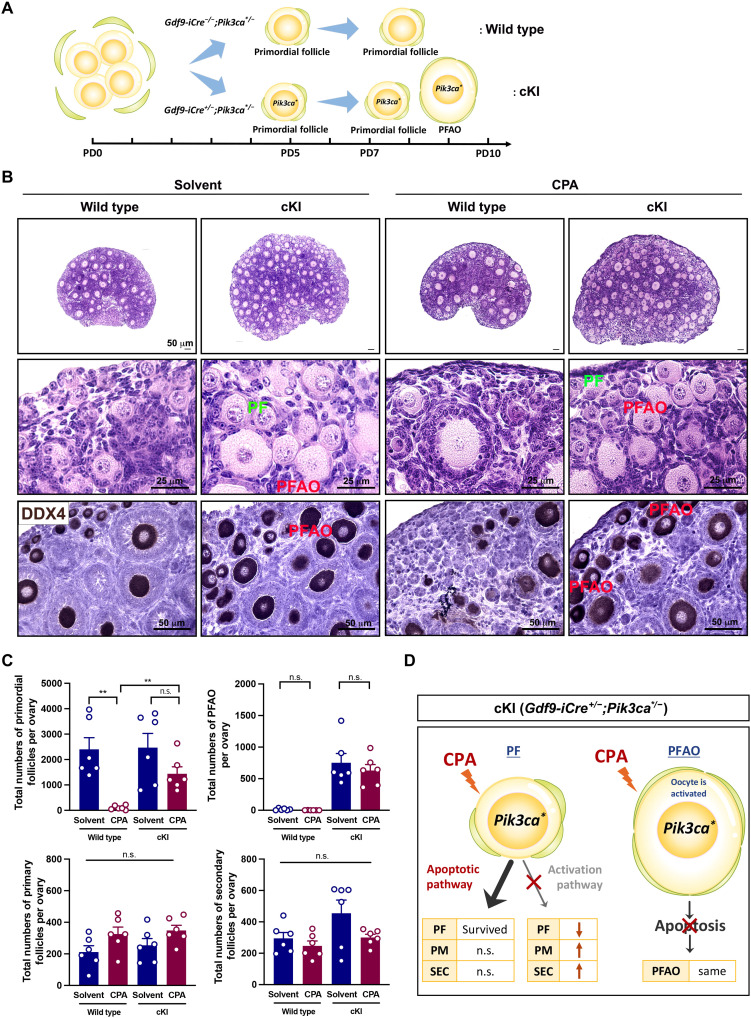
Oocytes with constitutive PI3K activity survived from CPA exposure. (**A**) Schematic of the status of primordial follicle (PF) and PFAO in wild-type or Pik3ca^*^ cKI mouse from PD0 to PD10. (**B**) Representative histology and 3,3-diaminobenzidine (DAB) staining with DDX4, an oocyte marker, of the ovaries from solvent or CPA-treated wild-type and Pik3ca^*^ cKI mice. Top: Hematoxylin and eosin (H&E)–stained whole ovarian histology. Scale bars, 50 μm. Middle: H&E staining with higher magnification of primordial follicle and PFAO. Scale bars, 25 μm. Bottom: Anti-DDX4 DAB staining of surviving oocytes. Scale bars, 50 μm. (**C**) Follicle quantification of solvent or CPA-treated wild-type and Pik3ca^*^ cKI mice (*n* = 6). n.s., not significant. **P <0.01. (**D**) Summary of CPA-induced consequences in follicle numbers from Pik3ca^*^ cKI mice. PM, primary follicle; SEC, secondary follicle.

A previous study suggested that CPA induces activation of primordial follicles via the phosphatidylinositol 3-kinase (PI3K)/PTEN (phosphatase and tensin homolog deleted on chromosome ten)/Akt pathway and increases the number of growing follicles (*9*). To evaluate whether oocytes of primordial follicles in dormant status are more sensitive to CPA, we generated transgenic Gdf9-iCre^+/−^; Pik3ca^*/−^ [Pik3ca^*^; conditional knockin (cKI)] mice in which PI3K was constitutively activated from oocytes of primordial follicles, meaning that the oocytes of primordial follicles have a high level of PI3K activity. As previously reported (*19*–*21*), the expression of PI3K induced the occurrence of PFAOs (primordial follicle with activated oocytes) in the ovary around PD7 (postnatal day 7), while the ovary of wild-type mice contained only normal primordial follicles (Fig. 1A). DDX4 (DEAD-box helicase 4 ) was used to indicate surviving oocytes because DDX4 is present in fetal and adult gonadal germ cells (*22*). The ovaries of PD7 wild-type and cKI mice treated with solvent or CPA (100 mg/kg) showed that more follicles survived in the ovaries of cKI mice than those of wild-type mice (Fig. 1B). The dose of CPA (100 mg/kg) was equivalent to the clinical dose in humans and was determined on the basis of the percentage of dead primordial follicles, which was tested using different doses of CPA (fig. S1, A and B). The ovaries of CPA-treated wild-type mice contained only primary and secondary follicles, whereas cKI mice preserved primordial follicles and PFAOs as well as growing follicles after CPA (Fig. 1B). Accordingly, the numbers of surviving primordial follicles and PFAOs in cKI mice were comparable between solvent and CPA-treated groups, while CPA depleted the primordial follicles of wild-type females. No significant changes were detected in the numbers of primary and secondary follicles in both wild-type and cKI groups after CPA treatment compared to solvent (Fig. 1C). The PI3K overexpression in oocytes preserved primordial follicles and PFAOs from CPA exposure (Fig. 1D), supported by more numbers of surviving primordial follicles and PFAOs compared to wild type. In addition, the total numbers of primary and secondary follicles were not affected with/without CPA exposure, implying that the primordial follicles were not activated by CPA. This indicates that the sensitivity to DNA damage is higher in oocytes of dormant status than in oocytes with high PI3K activity without increasing the number of growing follicles.

### ABL tyrosine kinase cannot be a target protein for protecting CPA-induced oocyte death in primordial follicles

**Fig. 2.**
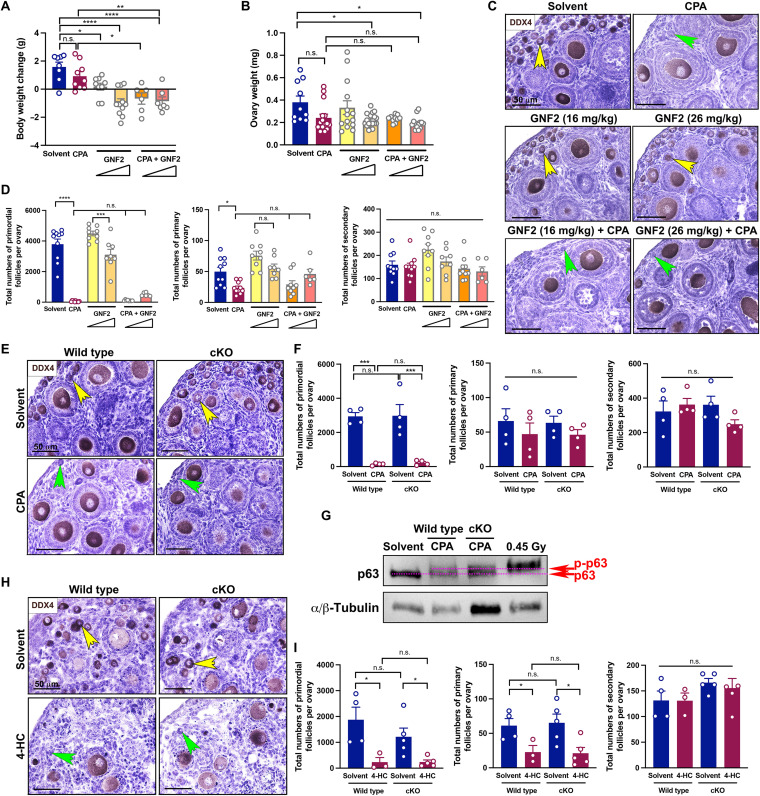
ABL tyrosine kinase was dispensable for primordial follicle loss by CPA. (**A**) Body weight changes of PD8 CD-1 mice exposed to solvent, CPA, GNF2, or cotreatment of GNF2 and CPA (*n* > 6). (**B**) Ovary weight of mice exposed to solvent, CPA, GNF2, or cotreatment of GNF2 and CPA. The ovarian weight of both sides in surviving mice was indicated with circles (*n* > 10). (**C**) Representative histology of ovaries from mice after treatment of solvent, CPA, GNF2, or cotreatment of GNF2 and CPA. DAB staining with DDX4 represents surviving oocytes. Yellow arrowheads, surviving primordial follicle; green arrowheads, follicle-like structure without oocyte. Scale bars, 50 μm. (**D**) Follicle quantification of both ovaries from each mouse after treatment of solvent, CPA, GNF2, or cotreatment of GNF2 and CPA. (**E**) DAB staining of the ovaries from wild-type and *Abl1* cKO mice treated with solvent or CPA. DAB staining with DDX4 represents surviving oocytes. Yellow arrowheads, surviving primordial follicle; green arrowheads, follicle-like structure without oocyte. Scale bars, 50 μm. (**F**) Follicle quantification of ovaries from wild-type and *Abl1* cKO mice treated with solvent or CPA (*n* = 4). (**G**) Immunoblotting analysis of the ovaries from wild-type and *Abl1* cKO mice treated with solvent or CPA. A 0.45-Gy irradiated ovary from a wild-type mouse was used for positive control. The p-p63 and p63 band positions are indicated by dotted lines. (**H**) DAB staining of the ovaries from wild-type and *Abl1* cKO mice cultured with solvent or 4-HC for 96 hours ex vivo. DAB staining with DDX4 represents surviving oocytes. Yellow arrowheads, surviving primordial follicle; green arrowheads, follicle-like structure without oocyte. Scale bars, 50 um. (**I**) Follicle quantification of the ovaries from wild-type and *Abl1* cKO mice treated with solvent or 4-HC ex vivo (*n* = 4). *P < 0.05; **P < 0.01; ***P < 0.001; ****P < 0.0001.

GNF2, a selective non–adenosine 5′-triphosphate–competitive inhibitor of BCR -ABL, is proposed as a fertoprotectant against CPA (*17*). To examine the functional efficacy of GNF2, the same concentrations of GNF2 as used in the previous report were administrated with CPA. Although body weight changes and ovary weights were comparable between the solvent and CPA groups, primordial follicles in the CPA group were rarely found in vivo (Fig. 2, A to C, and fig. S2B). Most of all, GNF2 did not present a protective effect against CPA regarding body weight, ovary weight, and surviving primordial follicles. GNF2 showed toxicity rather than protection when coadministered with CPA, as indicated by a lower survival rate and destructed ovaries (fig. S2). The follicle counting data confirmed that GNF2 did not prevent primordial follicle loss against CPA, whereas no significant changes in growing follicle numbers were noticed in vivo (Fig. 2D). However, a previous study proposed that GNF2 effectively prevented CPA-induced oocyte death by blocking ABL kinase in mouse ovaries (*17*). Thus, we genetically examined this discrepancy using oocyte-specific conditional knockout (cKO) of *Abl1* mice (*Gdf9-iCre^+/−^; Abl1 ^f/f^* ; cKO). In accordance with the GNF2 results, deletion of *Abl1* in the oocytes did not protect primordial follicles from CPA, whereas it preserved growing follicles (Fig. 2, E and F). Thus, ABL1 is dispensable for CPA-induced oocyte apoptosis. Consistently, CPA hyperphosphorylated TAp63 in the ovaries of the cKO mice as 0.45-Gy radiation did in the ovary of the wild-type mouse (Fig. 2G), suggesting that ABL kinase does not phosphorylate p63. To examine the direct effects of CPA, we used our well-established ex vivo culture system with 4-hydroperoxy-CPA (4-HC), a congener of CPA that can be converted into its key metabolite without enzymatic reactions. Consistent with the in vivo results, primordial follicles were susceptible to 4-HC in both ex vivo–cultured ovaries of wild-type and cKO mice, supported by histology and counting data of surviving follicles (Fig. 2, H and I). In summary, these findings elucidated that ABL tyrosine kinase is dispensable for CPA-induced primordial follicle loss, and targeting ABL with GNF2 has no protective effect from CPA.

### DNA damage checkpoint kinase 2 (CHK2)–TAp63 cascade is a key pathway to induce oocyte death of primordial follicles by CPA and DOXO

**Fig. 3.**
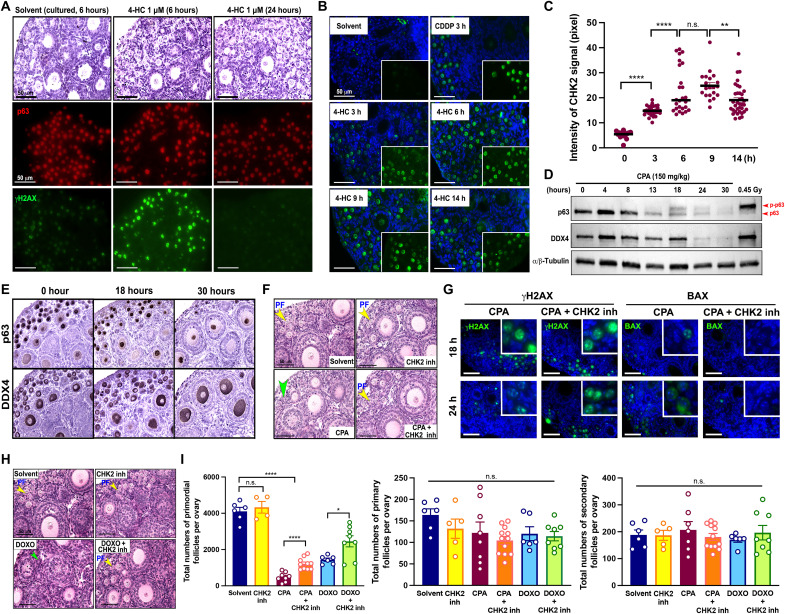
The CHK2-p63 signaling is the main pathway in the oocyte death of primordial follicles. (**A**) Time-dependent expression of p63 and γH2AX in the ovaries cultured with solvent or 4-HC at 6 and 24 hours ex vivo. Top: H&E staining. Middle: Immunofluorescence (IF) assay with p63 antibody. Bottom: IF assay with γH2AX. Scale bars, 50 μm. (**B**) IF assay of p-CHK2 on ovaries cultured with solvent or 4-HC for 3, 6, 9, and 14 hours. The CDDP-cultured ovary was used as a positive control. Insets represent images without 4′,6-diamidino-2-phenylindole overlay. Scale bars, 50 μm. (**C**) Intensity analysis of p-CHK2 signals at 0, 3, 6, 9, and 14 hours after culture with 4-HC. (**D**) Immunoblotting analysis of p63, DDX4, and α/β-tubulin in CD-1 mouse ovaries after CPA injection (150 mg/kg) at different time points. The wild-type CD-1 ovaries irradiated with 0.45 Gy were used as a positive control. (**E**) DAB staining with p63 and DDX4 of the paired ovaries after CPA injection at 0, 18, and 30 hours. (**F** and **H**) Representative histology of ovaries exposed to solvent, CHK2 inhibitor, CPA or DOXO (20 mg/kg), and cotreatment of CPA or DOXO (20 mg/kg) and CHK2 inhibitor. Yellow arrowheads, surviving primordial follicle; green arrowheads, follicle-like structure without oocyte. Scale bars, 50 μm. (**G**) IF assays of γH2AX (left) and BAX (right) expression in the ovaries harvested at 18 and 24 hours after CPA or CPA + CHK2 inhibitor injection. Scale bars, 50 μm. (**I**) Follicle quantification of ovaries collected from CD-1 mice 3 days after solvent, CHK2 inhibitor, CPA or DOXO, and cotreatment of CPA or DOXO and CHK2 inhibitor (*n* > 6). *P < 0.05; **P < 0.01; ****P < 0.0001.

It has been suggested that CPA can induce a unique death pathway in the oocytes of primordial follicles that differs from CDDP or radiation (*10*–*12*, *18*, *23*). To investigate the pathway triggered by CPA in oocytes, intensities of γH2AX, a DNA damage marker, and p-CHK2, a DNA damage responder, were measured in a timely manner. The expression of γH2AX was highly detected in oocytes at 6 hours in 4-HC–cultured ovaries and disappeared at 24 hours ex vivo, although the expression of p63 was consistent within 24 hours (Fig. 3A). The intensities of p-CHK2 timely changed, showing that the intensity was at its peak at 9 hours ex vivo (Fig. 3, B and C). Accordingly, the hyperphosphorylation of p63 in the ovary after CPA injection was observed at 18 hours as 0.45-Gy radiation hyperphosphorylated p63 in the ovary and decreased afterward in vivo, supported by the decrease of primordial follicles, showing fewer primordial follicles with the expression of p63 and DDX4 at 30 hours (Fig. 3, D and E). Furthermore, the pharmacological inactivation of CHK2 protein using the CHK2 inhibitor prevented the primordial follicle depletion caused by CPA, showing more surviving primordial follicles in the CPA + CHK2 inhibitor group than in the CPA group (Fig. 3, F and I, orange). However, the CHK2 inhibitor did not fully protect primordial follicles from CPA, implying that multiple factors such as the concentration of CHK2 inhibitor, the timing, and the kinetics of CHK2 and CPA would affect the consequence of follicle preservation. Furthermore, the CHK2 inhibitor did not prevent the CPA-induced DNA damage in primordial oocytes as the high expression of γH2AX was detected in both CPA and CPA + CHK2 inhibitor groups in vivo. However, the CPA-mediated downstream apoptosis was evaded by the CHK2 inhibitor as evidenced by the absence of BAX (Bcl-2–associated X; an apoptosis marker) expression in the oocytes (Fig. 3G).

DOXO is a topoisomerase inhibitor that blocks DNA duplication in actively proliferating tumor cells, whereas platinum-based CDDP and alkylating CPA induce DNA breaks via DNA binding (*24*–*28*). DOXO causes DNA double-strand breaks in a topoisomerase II–dependent manner or oxidative stress depending on cell types (*29*). Although oocyte genomes do not duplicate at the stage of primordial follicles, it has been proposed that DOXO directly induces DNA double-strand breaks in oocytes of primordial follicles of human and mouse ovaries, evidenced by the detection of histone H2AX phosphorylation and Ataxia telangiectasia mutated (ATM) activation (*30*). To examine whether CHK2 inhibitor can prevent loss of primordial follicles from DOXO, we coadministrated CHK2 inhibitor with DOXO because previous studies suggested that DOXO initiates primordial follicle death via the TAp63-related signaling pathway (*11*, *13*, *31*). Consistently, the CHK2 inhibitor preserved primordial follicles from DOXO, implying the importance of the CHK2-p63 pathway in the oocyte death of primordial follicles by DOXO (Fig. 3, H and I).

### p63 is a central regulator in oocyte elimination of primordial follicles by CPA and DOXO

**Fig. 4.**
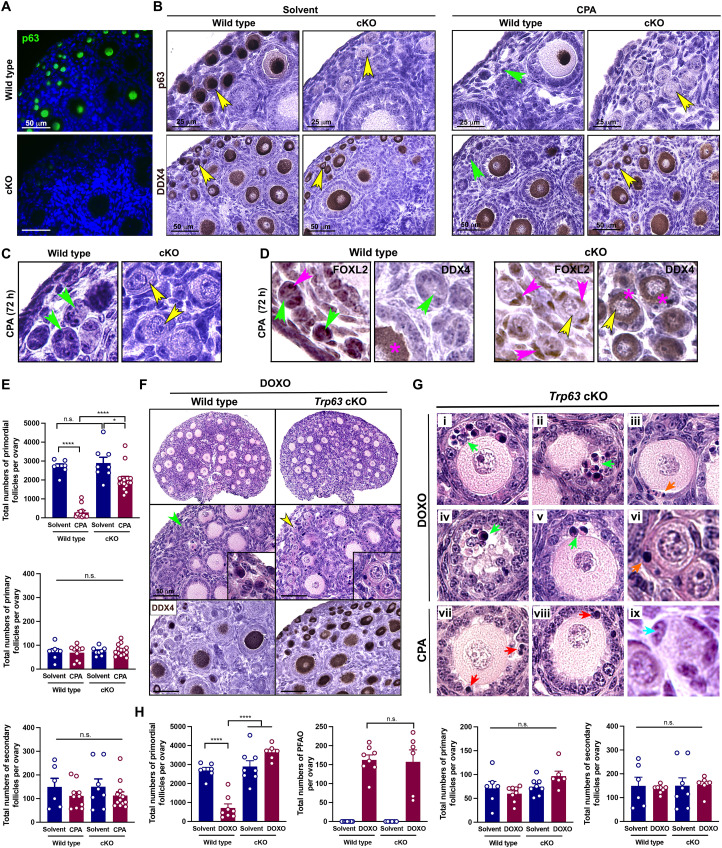
Knockout of Trp63 in oocytes preserves primordial follicles from cyclophosphamide and doxorubicin. (**A**) IF assays of p63 expression, proving oocyte-specific *Trp63* deletion in *Trp63* cKO mice. Scale bars, 50 μm. (**B**) DAB staining with p63 and DDX4 of the ovaries from mice treated with solvent and CPA. Yellow arrowheads, surviving primordial follicle; green arrowheads, follicle-like structure without oocyte. Scale bars, 50 μm. (**C**) Representative images with high magnification of the ovaries. Yellow arrowheads, surviving primordial follicle; Green arrowheads, follicle-like structure without oocyte. (**D**) High magnification of DAB staining with FOXL2 and DDX4 of the primordial follicles from wild-type and *Trp63* cKO mice treated with CPA. Green arrowheads, follicle-like structure without oocyte; yellow arrowheads, surviving primordial follicle; pink arrowheads, pregranulosa cells in follicle-like structure; pink asterisks, oocytes in surviving primordial follicles. (**E**) Follicle quantification of the ovaries from wild-type and *Trp63* cKO mice injected with solvent or CPA (*n* > 6). (**F**) Gross histology (top) of the ovaries from wild-type and *Trp63* cKO mice treated with DOXO. Representative histology (middle) of the ovaries with insets showing primordial follicles from each ovary. DAB staining with DDX4 antibody (bottom) of the ovaries from each genotype. Yellow arrowhead, surviving primordial follicle; green arrowhead, follicle-like structure without oocyte. Scale bars, 50 μm. (**G**) Representative histological images with high magnification of ovarian follicles from *Trp63* cKO treated with DOXO (i to vi) and CPA (vii to ix). (i, ii, iv, and v) Pointing out pyknotic granulosa cells with green arrowheads in secondary follicles; (iii) pyknotic pregranulosa cells of PFAO (orange arrowhead); (vi) pyknotic pregranulosa cells of primordial follicles (orange arrowhead); (vii and viii) pyknotic granulosa cells of secondary follicles (red arrowheads); (ix) healthy pregranulosa cells of primordial follicles (light blue arrowhead). (**H**) Follicle quantification of the ovaries from wild-type and *Trp63* cKO mice treated with solvent or DOXO (*n* = 6). *P < 0.05; ****P < 0.0001.

To further examine the role of p63 in oocyte death of primordial follicles by CPA administration, we generated oocyte-specific *Trp63* knockout mice and confirmed the absence of p63 expression in oocytes of primordial, primary, and early secondary follicles (Fig. 4A). The primordial follicles of *Trp63* cKO (*Gdf9-iCre^+/−^; Trp63 ^f/f^*) mice were resistant to CPA in vivo, while those of wild-type mice were absent in the ovary (Fig. 4B). Follicle-like structures of primordial follicles without oocytes were observed 72 hours after CPA injection in wild-type mice (Fig. 4C, green arrowheads). The primordial follicles with Forkhead box protein L2 (FOXL2)-positive pregranulosa cells (pink arrowheads) did not present the expression of DDX4 (green arrowheads) in the wild-type mouse ovary, confirming the oocyte depletion from primordial follicles by CPA. However, healthy primordial follicles with FOXL2 and DDX4 staining were observed in *Trp63* cKO (Fig. 4D and fig. S3). The quantification data confirmed that *Trp63* cKO mice prevented primordial follicles from CPA-induced primordial follicle loss (Fig. 4E). The importance of p63 in DOXO-induced oocyte apoptosis was also confirmed using the *Trp63* cKO mouse. The ovaries of *Trp63* cKO mice injected with DOXO (10 mg/kg) maintained healthy primordial follicles, exhibiting large numbers of surviving DDX4-positive oocytes in DOXO-treated *Trp63* cKO mice (Fig. 4F). DOXO-exposed ovarian follicles exhibited different phenotypes compared to CPA, showing apoptotic, multilayer granulosa cells of growing follicles (Fig. 4G, green arrowheads in i, ii, iv, and v); enlarged oocytes covered with a single layer of thin, squamous granulosa cells (Fig. 4G, orange arrowhead in iii); and pyknotic pregranulosa cells of surviving primordial follicles (Fig. 4G, orange arrowhead in vi). However, CPA induced pyknosis in only granulosa cells of growing follicles but not in pregranulosa cells of primordial follicles (Fig. 4G, vii to ix, red and blue arrowheads, respectively). The follicle counting data confirmed that the number of primordial follicles were preserved in the ovaries of *Trp63* cKO mice treated with DOXO (Fig. 4H). Together, the deletion of *Trp63* from oocytes prevented CPA- and DOXO-induced primordial follicle depletion, suggesting that p63 is an instrumental player in oocyte death of primordial follicles caused by CPA and DOXO.

### The absence of TAp63 evades the apoptotic pathway and directs the survival pathway in oocytes by CPA and DOXO

**Fig. 5.**
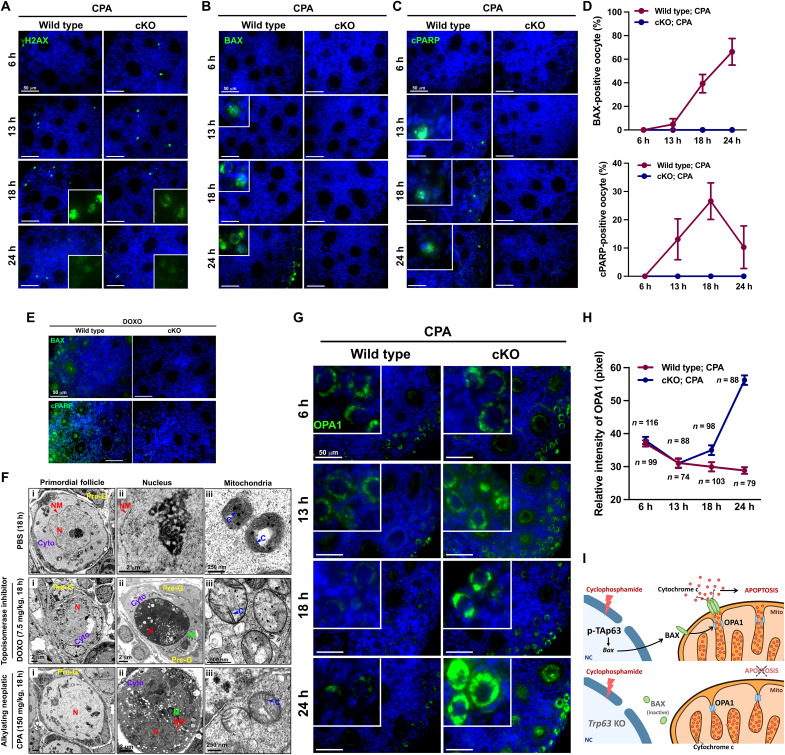
Knockout of *Trp63* in oocytes enhances the survival pathway rather than the apoptosis pathway. (**A** to **C**) IF assays of γH2AX (A), BAX (B), and cPARP (C) expression in the ovaries from wild-type and *Trp63* cKO female mice at 6, 13, 18, and 24 hours after CPA injection. Scale bars, 50 μm. (**D**) Percentage of primordial oocytes with BAX (top) and cPARP (bottom) expression. (**E**) IF assays of BAX (top) and cPARP (bottom) expression in the ovaries from wild-type and *Trp63* cKO mice treated with solvent or DOXO for 24 hours. Scale bars, 50 μm. (**F**) Transmission electron microscopy (TEM) images of gross primordial follicles, nucleus, and mitochondria from CD-1 mice at 18 hours after phosphate-buffered saline (PBS), DOXO, and CPA injection. NM, nucleus membrane; Pre-G, pregranulosa cell; N, nucleus; Cyto, cytoplasm; M, mitochondria; C, cristae of mitochondria. (**G**) IF assays of OPA1 expression in the ovaries from wild-type and *Trp63* cKO female mice at 6, 13, 18, and 24 hours after CPA injection. Insets show high magnification of primordial follicles with the expression of OPA1. Scale bars, 50 μm. (**H**) Relative intensity of OPA1 in primordial follicles of the ovaries from wild-type and *Trp63* cKO female mice at 6, 13, 18, and 24 hours after CPA injection. The numbers of oocytes measured for the intensity of OPA1 were counted. (**I**) Schematic of TAp63-regulated apoptosis in oocytes of primordial follicles.

Our previous study showed that the absence of *Trp63* suppressed the expression of CDDP-induced apoptosis-related molecules such as ABL, p53, and p73 (*10*), indicating that TAp63 in the oocytes of primordial follicles played a central role in controlling apoptotic pathways induced by gonadotoxic agents. Thus, to examine whether the deletion of *Trp63* from oocytes controls CPA-induced apoptosis as well, DNA damage–associated markers were examined in the ovaries of wild-type and *Trp63* cKO mice with CPA injection. CPA induced DNA damage in the ovaries of both wild-type and *Trp63* cKO mice, as evidenced by the detection of γH2AX signals in the oocytes at 18 hours. However, the intensity of γH2AX was less in the ovaries of *Trp63* cKO mice than in the ones of wild-type mice (Fig. 5A). The apoptosis markers BAX and cleaved poly(adenosine diphosphate–ribose) polymerase (cPARP) were detected in the ovaries of wild-type mice after CPA injection within 13 to 24 hours. However, these markers were undetectable in the ovaries of *Trp63* cKO mice within the same time window (Fig. 5, B to D). Consistently, BAX and cPARP were expressed in the ovaries of wild-type mice but not in the ones of *Trp63* cKO mice when DOXO was injected, suggesting that the absence of p63 suppressed the expression of DOXO-induced apoptosis in oocytes of primordial follicles (Fig. 5E).

To examine the toxic effect of CPA and DOXO on organelles in the oocytes of primordial follicles (*32*, *33*), transmission electron microscopy (TEM) images were examined after DOXO and CPA treatment (Fig. 5F). In primordial follicles at the stage of development, oocytes contained healthy mitochondria with cristae, nuclei filled with heterochromatin, Golgi apparatus, and lysosomes. Mitochondria in healthy oocytes remained organized in the cytoplasm, as shown in the phosphate-buffered saline–treated PD5 mouse ovary. However, DOXO and CPA induced necrotic and condensed nuclei in oocytes of primordial follicles, containing disorganized organelles, numerous lysosomes, and enlarged mitochondria with fewer cristae. Although DOXO and CPA have different working mechanisms to destroy tumor cells, they both cause condensed nuclei and swollen mitochondria in oocytes of primordial follicles.

To examine whether oocytes of primordial follicles with the absence of TAp63 block the apoptotic pathway in the condition of damaged oocytes or enhance the survival pathway, we further assessed the expression of mitochondrial-related apoptosis marker optical nerve atrophy 1 (OPA1), which regulates the release of cytochrome c. The expression of OPA1 gradually decreased in the ovaries of CPA-treated wild-type mice in a time-dependent manner (Fig. 5, G and H). The intensity of the OPA1 signal was gradually increased within this time window after CPA administration in the ovaries of *Trp63* cKO (Fig. 5, G and H), up-regulating the mitochondrial dynamic activities in the oocytes treated with CPA. Thus, CPA induces cytochrome c–dependent apoptosis controlled by phospho-TAp63-BAX-OPA1 in the oocytes of wild-type mice. Meanwhile, the absence of *Trp63* impeded BAX activation to target the OPA1–cytochrome c release apoptotic pathway, although the mechanism underlying the increase of OPA1 by CPA in *Trp63* cKO remains to be further investigated (Fig. 5I).

### Primordial oocytes from *Trp63* knockout mice administrated with CPA are capable of normal fertility

**Fig. 6.**
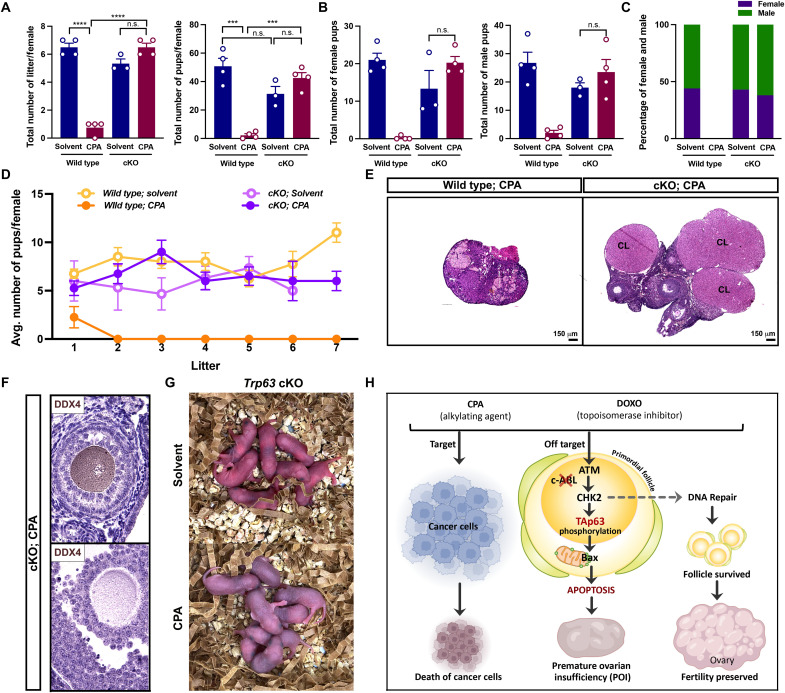
*Trp63* KO is sufficient to restore fertility against CPA. (**A**) The total number of litters and pups per female of wild-type and *Trp63* cKO female mice were counted after the continuous fertility test (*n* = 4). (**B**) The total number of female pups per litter (left) and the number of male pups per litter (right) were counted. (**C**) The percentage of female (purple) and male offspring (green) of wild-type and *Trp63* cKO female mice injected with solvent and CPA was calculated after the continuous fertility test (*n* > 3). (**D**) The average number of pups per female (*n* = 4) was monitored for 5 months from the day of mating. (**E**) Ovarian H&E histology of wild-type and *Trp63* cKO female mice after fertility test. CL, corpus luteum. Scale bars, 150 μm. (**F**) DAB staining with DDX4 of the secondary follicle (top) and antral follicle (bottom) from *Trp63* cKO female mice treated with CPA after fertility test. (**G**) Images of pups born from *Trp63* cKO female mice injected with solvent (top) and CPA (right) during continuous fertility test. (**H**) Graphical summary of the strategy to target TAp63-related signaling pathway in the clinical setting using CPA and DOXO for preserving ovarian reserve. ***P < 0.001; ****P < 0.0001.

To examine whether oocytes from *Trp63* cKO mice survived, overcame DNA damage, and contributed to producing normal offspring after CPA administration, cKO mice with/without CPA treatment were mated with fertility-proven males to test their reproductive function. Female mice were treated with CPA (100 mg/kg) twice at PD7 and PD28 for both wild-type and cKO groups to deplete primordial follicles before mating. Notably, the total litter numbers and pup numbers in cKO mice injected with CPA were indistinguishable from those of solvent-treated cKO, differing from CPA-treated wild-type mice (Fig. 6A). There is no difference between genders regarding the total pup number (Fig. 6B). Most of all, the reproductive function of cKO/CPA females was maintained until the end of the fertility test and comparable with females in the wild-type/solvent- and cKO/solvent-treated groups (Fig. 6D). Wild-type/CPA females stopped reproducing after delivering one litter (Fig. 6D, closed orange circle). At 5 months after mating, ovarian histology showed a marked difference between wild type and cKO with CPA treatment. While the ovaries of cKO mice had multiple corpus lutea and growing follicles, the ovaries of wild-type mice showed aged phenotypes with shrunken size (Fig. 6E). The expression of DDX4 in the secondary follicle and antral follicle indicates the normal progression of folliculogenesis in the ovary of cKO mice treated with CPA (Fig. 6F). The offspring from CPA-injected cKO mice appeared normal in morphology compared to the solvent-injected group, indicating that oocytes rescued from CPA-induced apoptosis are viable (Fig. 6G). Thus, our results demonstrated that TAp63 in the oocyte nucleus is instrumental in determining the apoptosis of primordial follicles induced by gonadotoxic chemotherapies, and the blockage of TAp63 signaling is the key step for preserving ovarian reserve (Fig. 6H).

## DISCUSSION

Because chemotherapeutic agents are well established in clinical practice, it is of immense value to understand not only how these treatments target cancer cells but also how they affect ovarian reserve and consequently affect reproductive life span. It was proposed that CPA treatment induces primordial follicle loss via phosphatidylinositol kinase (PI3K)/PTEN/AKT pathway, as CPA affected growing follicles and lowered AMH production, which consequently increased the pool of growing follicles from the ovarian reserve (*9*). The concept of the primordial follicle pool overactivation was supported by increased Akt/mTOR expression in immunoblotting analysis with whole ovarian extracts rather than oocytes isolated from CPA-exposed primordial follicles. However, our previous study specifically detected apoptosis markers in primordial follicles after CPA injection in the same experimental condition (*12*). Moreover, other studies also found that CPA induces DNA damage and apoptosis in the oocytes of primordial follicles (*34*). To further confirm this, we took advantage of the Pik3ca^*^ cKI mouse, which expresses PI3K constitutively in oocytes from the stage of primordial follicles. Results using the Pik3ca^*^ cKI mouse model presented that the ovaries from Pik3ca^*^ cKI mouse injected with CPA contain more surviving primordial follicles and PFAOs because of PI3K activity, indicating that oocytes of dormant status have a high sensitivity to DNA damage, while oocytes with activating signals similar to oocytes of primary follicles or oocytes with PI3K activity are not sensitive enough to apoptosis by DNA damage. Our study indicates that primordial follicles with normal physiological level of PI3K activity die via apoptotic pathway rather than being activated as proposed in the burnout theory. Accordingly, a recent transcriptomic study performed single-cell RNA sequencing on oocytes of only primordial follicles after CPA injection in a human ovarian xenograft model to dissect the pathway of oocyte depletion. The individual oocyte transcriptomic analysis exhibited a significant decrease in the expression of the antiapoptotic pathway including *pro-Akt*, platelet endothelial cell adhesion molecule 1 (*PECAM1*), *IKBKE,* and *ANGPT1* and reduced activation of PI3K/PTEN/Akt after CPA exposure (*35*), implying that primordial follicles in human ovarian tissue do not turn on activation signals in response to the acute effects of CPA. Meanwhile, both γH2AX and cleaved caspase-3 were detectable in primordial follicles, especially Bcl-2–associated death promoter (BAD). These findings signify that the depletion of ovarian reserve induced by CPA is based on the apoptotic mechanism.

The role of ABL in regulating chemotherapy-induced apoptosis in oocytes remains debated. Although evidence suggested that ABL inhibitors protect the ovarian reserve from CDDP-induced degeneration (*36*), genetic studies confirmed that ABL is dispensable for regulating oocyte death by CDDP (*11*). It has also been proposed that ABL is not necessary for CDDP-induced oocyte death and is not crucial for p63-mediated apoptosis in the human ovary (*37*). As for CPA, the previous study showed that cotreatment of CPA and ABL inhibitor, GNF2, preserved primordial follicles from CPA-induced DNA damage (*17*). However, the administration of GNF2 did not protect primordial follicle loss from CPA in our study. Furthermore, our study using the *Abl1* cKO mouse model confirmed that ABL is dispensable for oocyte death of primordial follicles by CPA and is not involved in p63-regulated apoptosis. However, this does not exclude the possibility that GNF2 inhibits ABL in pregranulosa cells to transfer the surviving signals to oocytes of primordial follicles. In contrast, our study proposes that the CHK2 inhibitor rather than GNF2 can be used as a fertoprotectant candidate because it prevented the primordial follicle loss induced by CPA, CDDP, DOXO, and irradiation (*11*, *38*–*40*). We expected the CHK2 inhibitor to completely rescue primordial follicles in CPA-treated mice, but the CHK2 inhibitor only partially protected primordial follicles from CPA exposure. We assume that multiple factors such as the concentration of CHK2 inhibitor, the timing, and the kinetics of CHK2 and CPA would affect the consequence of follicle preservation. CHK2 inhibitor prevented the CPA-induced apoptosis but not DNA damage in the oocytes, as evidenced by the loss of BAX expression and the presence of γH2AX after CPA and CHK2 inhibitor cotreatment. This indicates that DNA damage resides in the genome of oocytes without repair. Although the CHK2 inhibitor has a protective effect on oocyte apoptosis against CPA, the potential for using CHK2 inhibitor as a fertoprotectant requires further study as it may detour DNA repair and impair cell cycle regulation.

The transcription factor p63, one of the p53 family members, plays an essential role in determining oocyte death and regulating maternal reproduction, genomic integrity, and epidermal development (*11*, *13*, *41*, *42*). Although the previous report suggested that TAp63 is not related to oocyte death by CPA (*18*), our study confirmed that CPA hyperphosphorylates TAp63 in a time-dependent manner and revokes oocyte death of primordial follicles in *Trp63* cKO mice. The deletion of p63 in oocytes also abolished the CPA-stimulated apoptosis pathway in primordial follicles, exhibiting no apoptosis signals although DNA damage occurred. Most of all, the reproductive potential of the surviving primordial follicles from *Trp63* cKO ovaries was also confirmed by a fertility test showing comparable litter sizes and numbers in CPA-treated *Trp63* cKO mice versus solvent-treated mice. A recent study from another group suggested that oocytes efficiently repaired DNA double-strand breaks induced by radiation and CDDP through the homologous recombination pathway (*43*, *44*). However, a recent transcriptome analysis of oocytes following irradiation could not detect the up-regulation of DNA repair programs, indicating that oocyte fate is decided by the intensity of the apoptotic signals instead of DNA repair pathways (*45*). Nonetheless, the genotoxic intensity of direct irradiation is more acute and lethal than typical chemotherapeutic agents. In the face of such intense genotoxic stress, DNA repair mechanisms are insufficient to eliminate genotoxicity. However, chemotherapeutic agents accumulate in oocytes and gradually induce DNA damage, raising the importance and possibility of DNA repair in response to chemotherapy. ATM, Ataxia telangiectasia and Rad3-related protein (ATR), and DNA-Protein kinase C (PKcs) are considered to be the controlling factors of double-strand DNA breaks in mammals. They are recruited to and activated at the DNA damage sites and then phosphorylate downstream proteins including CHK2 to facilitate DNA repair. Thus, further studies are required to understand whether these DNA repair proteins are activated to restore DNA integrity and prevent genetic abnormality of offspring from females exposed to chemotherapeutic agents on a whole-genome level (*46*–*49*).

In addition, DOXO also triggered oocyte death in primordial follicles through a TAp63-related pathway as consistent with a previous report (*31*). The deletion of p63 rescued the primordial follicles and evaded the apoptosis pathway in oocytes against DOXO treatment, supporting the previous report that DOXO directly induces DNA double-strand breaks and activates ATM, which stimulates TAp63 phosphorylation (*30*). Our study also noticed that DOXO caused pyknosis in granulosa cells of growing follicles without affecting the oocytes, as corroborated by a previous study demonstrating that granulosa cells were more sensitive to DOXO as evidenced by earlier and higher expression of DNA damage compared to oocytes (*29*). This is due to the rapid division and DNA replication of granulosa cells, supporting DOXO-induced DNA damage in granulosa cells. In addition, pyknosis was observed in nonproliferating pregranulosa cells in surviving primordial follicles of *Trp63* cKO after DOXO treatment, implying that the oocyte survival of primordial follicles does not seem to be determined by pyknosis in pregranulosa cells. This has not been observed in ovarian primordial follicles from CDDP-, CPA-, and radiation-treated mice. Thus, we speculate that DOXO seems to be more accumulated in pregranulosa/granulosa cells.

OPA1 expression gradually increased in *Trp63* cKO oocytes in primordial follicles 24 hours after CPA treatment, whereas its expression declined before oocytes were eliminated in the wild-type ovaries treated with CPA. OPA1 is a dynamin-related protein located in the inner mitochondrial membrane essential for mitochondrial fusion (*50*, *51*). More evidence suggests that OPA1 mediates cristae structure remodeling and regulation of apoptosis through the compartmentalization of soluble cytochrome c within the cristae independent from the fusion process (*51*, *52*). The activation of TAp63 by CPA-induced DNA damage up-regulates BAX, which permeabilizes the mitochondrial outer membrane resulting in cytochrome c release (*53*, *54*). The oligomerized BAX also triggers the disassembly of OPA1 that holds cytochrome c within cristae, which accelerates the apoptosis progression (*55*–*57*). This is correlated with the fact that the ovaries of CPA-treated *Trp63* cKO mice demonstrated undetectable expression of BAX or cPARP, which prevented oocyte loss, suggesting that the presence of functional TAp63 is critical for inducing apoptotic pathway in the oocytes. Nevertheless, how oocytes without *Trp63* stimulate OPA1 expression upon CPA-induced DNA damage remains unclear. Furthermore, whether p63 directs the mitochondrial self-repair requires further study.

This study clarified the underlying mechanism of CPA and DOXO in oocyte death and provided basic knowledge for developing fertoprotective agents to preserve ovarian follicles in patients with cancer. In this study, we emphasize that primordial follicles are directly affected by CPA treatment without being activated, suggesting that dormant follicles are more sensitive to CPA compared to growing follicles. In addition, TAp63 is the master regulator to induce follicle depletion via DNA damages caused by alkylating agents (e.g., CPA) and topoisomerase inhibitors (e.g., DOXO). Our data specified that the CHK2-TAp63 pathway rather than ABL-DNA-PK-TAp63 is the main pathway in oocyte death of primordial follicles induced by chemotherapeutic agents. For future studies, a screening of reproductive health from patients with triple-negative breast cancer enrolled in clinical trials (ClinicalTrials.gov identifier: NCT02203513) using the CHK1/2 inhibitor (LY2606368) is necessary to clarify the gonadotoxicity of CHK2 inhibitor in humans. Although our studies show that CHK2 inhibitor protects primordial follicles from chemotherapy and radiation therapy, it is hard to suggest CHK2 inhibitor as an ideal fertoprotectant to preserve ovarian reserve as it may affect DNA damage surveillance in the oocytes. Because of the importance of TAp63 in determining oocyte fate against gonadotoxic chemotherapies, fertoprotectants targeting TAp63 should be developed to protect ovarian follicles and preserve ovarian function without compromising the efficacy of chemotherapy.

## MATERIALS AND METHODS

### Animal

CD-1 and C57BL/6 mice were purchased from Charles River Laboratories. *Gdf9-iCre* mice were purchased from the Jackson Laboratory. Mice carrying a floxed allele for Pik3ca^*^, *Abl1*, or *Trp63* were generated as previously described (*10*, *11*, *19*, *58*). All procedures involving mice were approved by the Institutional Animal Care and Use Committee at the University of Nebraska Medical Center (UNMC). Animals were provided with food and water ad libitum and kept in the Comparative Medicine facilities of UNMC. Temperature, humidity, and photoperiod (10 of 14 photoperiods) were kept constant.

Oocyte-specific *Abl1* and *Trp63* cKO mice were generated by breeding homozygous female mice with heterozygous *Gdf9-iCre^+/−^* male. The genotyping polymerase chain reaction conditions were generated as previously described (*10*, *11*, *19*). Transgenic mice with oocyte-specific expression of constitutively active PI3K (Pik3ca^*^) were generated by breeding the homozygous female with Pik3ca^*^ with heterozygous *Gdf9-iCre^+/−^* male. Gdf9-iCre^−/−^; Pik3ca^*/w^ and *Gdf9-iCre^+/−^*; Pik3ca^*/w^ mice were designated to wild type and cKI mice, respectively. *Gdf9-iCre^−/−^* (with *Abl1 ^f/f^* or *Trp63 ^f/f^*) and *Gdf9-iCre^+/−^* (with *Abl1 f/f* or *Trp63 f/f*) were designated to wild-type and cKO mice, respectively.

CD-1 females were injected on PD5 with solvent and CPA (75, 100, or 150 mg/kg; PHR1404, Sigma-Aldrich). CD-1 PD8 or PD10 mice were injected with GNF-2 (G9420, Sigma-Aldrich) or CHK2 inhibitor (CHK2 inhibitor II hydrate, C3742, Sigma-Aldrich), respectively, 2 hours before CPA administration and harvested after 3 days. The wild-type, Pik3ca^*^ cKI, *Abl1* cKO, and *Trp63* cKO mice were injected on PD7 with solvent, CPA, or DOXO (NDC 0069-3031-20, Pfizer), and ovaries were harvested after 3 days.

### Whole-organ ovary culture ex vivo

Whole ovarian organ culture was performed as previously described (*10*, *19*). The ovaries collected from PD5 CD-1, PD7 wild-type, and *Abl1 ^f/f^* cKO female mice were isolated from the ovarian bursa. Then, the ovaries were cultured on 0.4-μm Millicell cell culture inserts (PICM01250, MilliporeSigma) with a culture medium composed of minimum essential medium α (32571036, Gibco) supplemented with bovine serum albumin (1 mg/ml; BP9703100, Thermo Fisher Scientific) and insulin-transferrin-selenium (5 μg/ml; 41400045, Gibco). The ovaries were treated with 0 or 1 μM 4-HC (19527, Cayman Chemical Company) and harvested at 3, 6, 9, 14, or 96 hours.

### Follicle counting

The ovaries were fixed in modified Davidson’s fixative (3600, EKI) and serially sectioned at 5-μm thickness. The sections were stained with hematoxylin and eosin for histological analysis. The counting and calculating of each class of ovarian follicles were performed as previously described (*19*). PFAO was defined with an intermediate-sized oocyte (between 320 and 500 μm^2^) and very thin squamous single-layer granulosa cells in the morphological analysis (*59*).

### Immunofluorescence assays and 3,3-diaminobenzidine staining

The immunofluorescence (IF) and 3,3-diaminobenzidine (DAB) staining were performed as previously described (*10*, *19*). The Metal Enhanced DAB Substrate Kit (34065, Thermo Fisher Scientific) was used for DAB staining. The Alexa Fluor 488 Tyramide SuperBoost Kit (B40932, Invitrogen) was used for IF staining. The catalog numbers and dilution of primary antibodies were as follows: p63 (D9L7L) (39692s; 1:100), γH2AX (9718S; 1:100), cPARP (9548s; 1:50), p-CHK2 (2197s; 1:50), and OPA1 (D7C1A) (67589s; 1:50) from Cell Signaling Technology; BAX (p-19) (sc-526; 1:50) from Santa Cruz Biotechnology Inc.; and DDX4 (ab270534; 1:100) and FOXL2 (ab246511; 1:50) from Abcam. The intensity of p-CHK2 and OPA1 signals was measured by ImageJ using at least five random nonoverlapping images under the same exposure condition with quadruple experiments. The numbers of cPARP- and BAX-positive oocytes were measured by counting positive signals in at least five random nonoverlapping images by ImageJ with triplicate experiments.

### Immunoblotting

Ovaries harvested from CD-1 PD5 mice were flash-frozen with liquid nitrogen at 0, 4, 8, 13, 18, 24, and 30 hours after CPA injection (150 mg/kg). The whole ovaries were homogenized in Pierce radioimmunoprecipitation assay buffer (89900, Thermo Fisher Scientific) with protease inhibitor (11836153001, Roche Diagnostics GmbH) and phosphatase inhibitor cocktails (04906837001, Roche Diagnostics GmbH). Proteins were loaded into the 4 to 15% Mini-PROTEAN TGX Precast Protein Gels (#4561084, Bio-Rad Laboratories Inc.) and transferred to a nitrocellulose membrane using the Trans-Blot Turbo Transfer System (#1704150, Bio-Rad Laboratories Inc.). The blots were probed with primary antibodies, followed by secondary antibodies. The catalog numbers and dilution of primary antibodies were as follows: p63-α (D2K8X) (13109s; 1:1000) and α/β-tubulin (2148s; 1:2000) from Cell Signaling Technology and DDX4 (ab270534; 1:2000) from Abcam. Proteins were detected using the Clarity Western ECL Substrate (#1705061, Bio-Rad Laboratories Inc.) and exposed using the iBright CL1500 Imaging System (A44114, Invitrogen).

### Transmission electron microscopy

PD5 CD-1 female mice were injected once with solvent, CDDP (5 mg/kg), DOXO (7.5 mg/kg), and CPA (150 mg/kg) for 18 hours of treatment, followed by further TEM steps. The preparation of ovarian sections was performed as previously described (*23*). Sections were examined on an FEI Tecnai G2 TEM operated at 80 kV after staining with 1% uranyl acetate and Reynold’s lead citrate.

### Fertility test

Wild-type and *Trp63* cKO female mice were injected twice on PD7 and PD28 with CPA (100 mg/kg) or solvent to remove the remaining primordial follicles. At PD42, females were subjected to fertility testing with fertility-proven C57BL/6 males. The reproductive activity of female mice was monitored for 5 months from the day of mating by recording the total number of litters and pups. The fertility test was stopped when CPA-treated females did not deliver pups for 3 months. The ovaries were harvested from those female mice for further analysis.

### Statistical analysis

Graphs were generated by Prism 9.1.1 software (GraphPad Software version 9), and data were presented as means ± SEM. One-way analysis of variance (ANOVA) with Tukey’s post hoc test was used to determine the statistical difference among groups. Unpaired two-tailed Student’s *t* test was used for paired comparison between groups. *P* values of less than 0.05 were considered statistically significant. n.s. represents not significant, and *, **, ***, and **** represent *P* < 0.05, *P* < 0.01, *P* < 0.001, and *P* < 0.0001, respectively.

## Notes

View/request a protocol for this paper from *Bio-protocol*.
