# Supplementary material for: TAp63 determines the fate of oocytes against DNA damage
Source: Sci Adv. 2022 Dec 21;8(51):eade1846. doi: 10.1126/sciadv.ade1846 (PMC9770984; doi:10.1126/sciadv.ade1846)
Supplement: Supplementary file 1 — Figs. S1 to S3 [file sciadv.ade1846_sm.pdf]

Science Advances
AAAS

Supplementary Materials for

TAp63 determines the fate of oocytes against DNA damage

Yi Luan *et al.*

Corresponding author: So-Youn Kim, soyoun.kim@unmc.edu

Sci. Adv. **8**, eade1846 (2022)
DOI: 10.1126/sciadv.ade1846

This PDF file includes:

Figs. S1 to S3

Figure S1

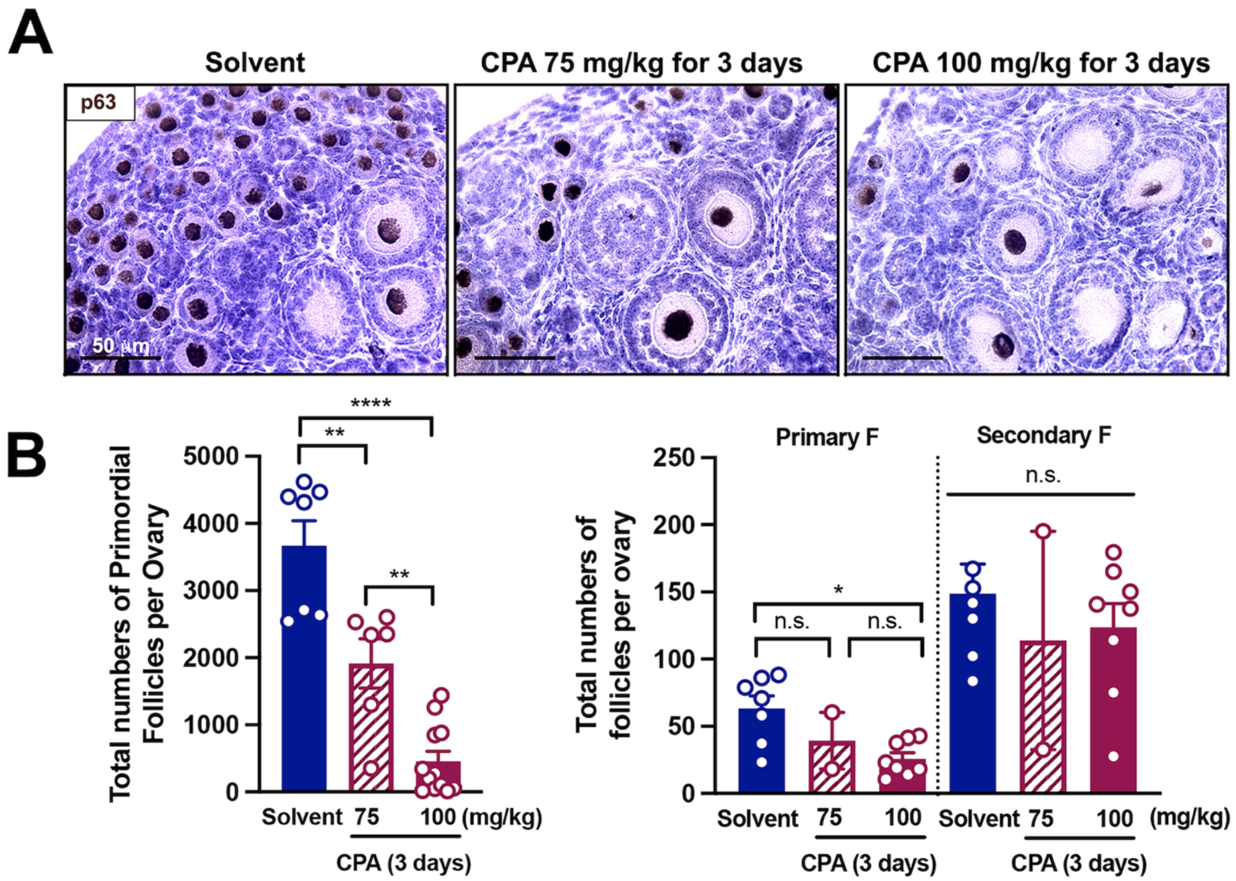

Fig. S1. CPA induces primordial follicle loss dose-dependently. (**A**) DAB staining with p63 of the ovaries from PD8 CD-1 mice treated 0, 75 mg/kg, and 100 mg/kg CPA for 3 days. Scale bar = 50 um. (**B**) The total numbers of primordial, primary, and secondary follicles of the ovaries from CD-1 mice at 3 days following 0, 75 mg/kg, and 100 mg/kg CPA injection (n > 6).

Figure S2

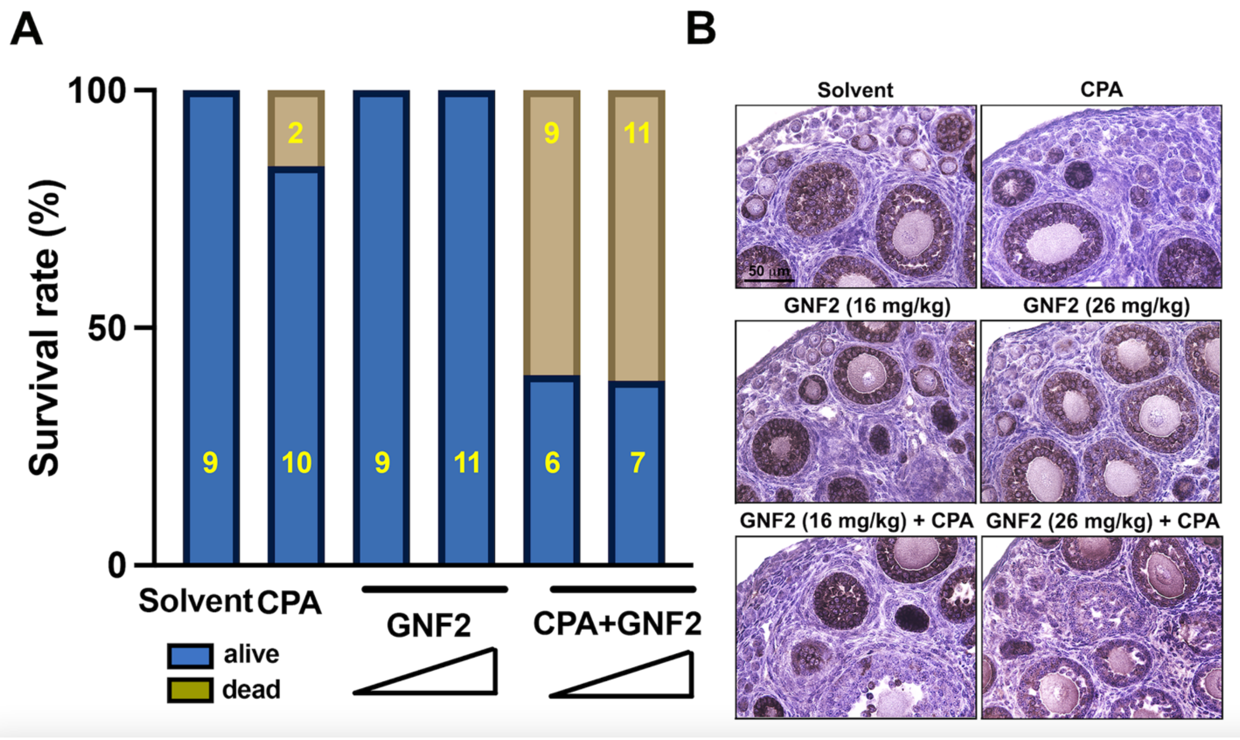

Fig. S2. GNF2 displays toxicity instead of protection when co-administrated with CPA. (**A**) The survival rate of CD-1 mice treated with solvent, CPA, GNF2, and co-treatment of CPA and GNF2. The number on the bar indicates the number of mice in each set of injections. (**B**) DAB staining with AMH expression in the ovaries from CD-1 mice after treatment of solvent, CPA (100 mg/kg), GNF2 (16 mg/kg and 26 mg/kg), and co-treatment of GNF2 and CPA. Scale bar = 50 um.

Supplemental Figure 3

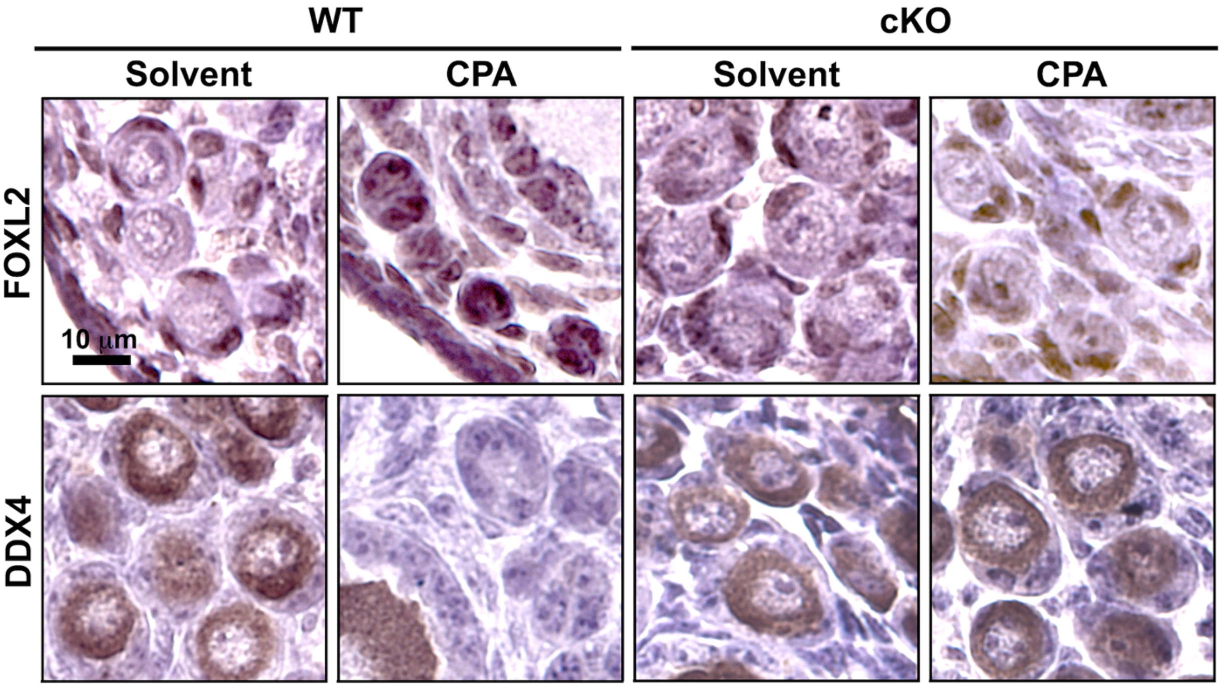

Fig. S3. CPA-induced oocyte depletion from primordial follicles in wildtype rather than *Trp63* KO mice. DAB staining with FOXL2 and DDX4 of the primordial follicles from wild-type and *Trp63* cKO female mice treated with solvent or CPA. Scale bar = 10 um.
